# lncRNA-MEG3 Suppresses the Proliferation and Invasion of Melanoma by Regulating CYLD Expression Mediated by Sponging miR-499-5p

**DOI:** 10.1155/2018/2086564

**Published:** 2018-04-02

**Authors:** Jianwen Long, Xianming Pi

**Affiliations:** ^1^Department of Dermatology, The First Clinical School, Hubei University of Chinese Medicine, Wuhan 430061, China; ^2^Department of Dermatology, Hubei Provincial Hospital of Traditional Chinese Medicine, Wuhan 430061, China

## Abstract

The abnormal expression of long noncoding RNA- (lncRNA-) MEG3 was clearly identified in a number of malignant tumors, but the specific function of MEG3 remains unknown in malignant melanoma until now. The research attempts to explore the effects of MEG3 on the growth and metastasis of malignant melanoma. MEG3 and miR-499-5p expression were determined by qRT-PCR method. Western blotting assay was applied to detect protein expression. Luciferase reporter assay was used to assess the correlation between MEG3 and miR-499-5p and between CYLD and miR-499-5p. Cell growth, cell cycle, and cell apoptosis were examined by CCK-8 assay, EdU assay, and flow cytometry assay, respectively. The invasion ability of melanoma cells was investigated by wound-healing and Transwell assays. The effect of MEG3 on growth of melanoma in vivo and cell chemosensitivity was detected by xenograft animal model and CCK-8 assay. As a result, the expression of MEG3 was decreased in melanoma tissues and cell lines. The level of MEG3 was significantly associated with poor prognosis. MEG3 could bind to miR-499-5p and CYLD mRNA contained a binding site of miR-499-5p. The expression of CYLD was reduced and the level of miR-499-5p was elevated in melanoma tissues and cell lines. Luciferase reporter assay and western blot assay confirmed that MEG3 regulated the expression of CYLD by sponging miR-499-5p. Functionally, upregulation of MEG3 inhibited melanoma cell proliferation, invasion, and migration, enhanced melanoma cell apoptosis, arrested melanoma cell cycle, and regulated the expression of E-cadherin, N-cadherin, and cyclin D1 by regulating CYLD expression mediated by sponging miR-499-5p. Importantly, overexpression of MEG3 suppressed the growth of xenograft tumor and improved chemotherapy sensitivity of A375 cells to cisplatin and 5-FU treatment. In conclusion, MEG3 has a crucial function in the tumorigenesis of melanoma, and MEG3 may be a potential therapeutic target in the treatment of melanoma.

## 1. Introduction

Malignant melanoma originating from melanocytes is an aggressive skin cancer which is listed as the seventh most frequent malignant tumor in females and the fifth most frequent malignant tumor in males worldwide [1]. The incidence of melanoma has been remarkably increasing in recent years, and the progression and metastasis of melanoma are extremely rapid [2, 3]. Accordingly, it is reasonably necessary to create innovative strategies for treating melanoma, and it may be hopeful to uncover the molecular mechanism of melanoma growth and metastasis for finding new therapeutic targets. Increasing evidences emphasized that lncRNAs played essential roles in regulating numerous biological activities including cell proliferation [4], cell autophagy [5], and tumor metastasis [6]. The dysregulation of lncRNAs has been considered to be related to the occurrence, development, and relapse of tumors [7]. Maternally expressed gene 3 (MEG3) is positioned on human chromosome 14q32.3 and encoded by a maternally imprinted gene [8]. Recent researches have demonstrated that MEG3 was involved in the tumorigenesis of diverse malignant tumors. For instance, overexpression of MEG3 suppressed significantly chronic myeloid leukemia cell growth and invasion by regulating the expression of miR-184 [9], MEG3 inhibited breast cancer cell growth and metastasis by blocking AKT pathway [10], and MEG3 limited EMT processing of breast cancer cell through regulating E-cadherin expression mediated by targeting miR-421 [11]. For all that, the main function of MEG3 on the development of melanoma remains remarkably obscure.

CYLD is a mutated gene found in familial cylindromatosis. Various reports have indicated that CYLD plays an antitumor role in numerous malignant tumors by regulating various signaling pathways, including Wnt/*β*-catenin [12], nuclear factor-*κ*B [13], and transforming growth factor-*β* [14]. CYLD has been identified to be downregulated in melanoma tissues and cell lines and to suppress melanoma cell proliferation and metastasis by blocking the JNK/AP-1 and *β*1-integrin signaling pathways [15].

MicroRNA is a short ribonucleotide acid sequence that can regulate gene expression by binding to the target mRNA [16]. More and more researches have manifested that microRNAs participate in the occurrence and development of many tumors [17]. MiR-499-5p was found to inhibit lung cancer growth and invasion via regulating the expression of VAV3 [18]. Conversely, some evidence manifested that miR-499-5p promoted colorectal cancer cell proliferation through inhibiting the expression of FOXO4 and PDCD4 [19]. Roles of miR-499-5p in melanoma were not still elucidated until now.

Bioinformatics analysis tool sheds important light on the relationship among MEG3, CYLD, and miR-499-5p. In this report, the expression and function of MEG3, miR-499-5p, and CYLD were explored in melanoma tissues and cell lines. The combined results identified that MEG3 suppressed melanoma cell proliferation and invasion by regulating the expression of CYLD mediated by sponging miR-499-5p.

## 2. Materials and Methods

### 2.1. Clinical Specimens and Cell Culture

The research was identified by the Ethics Committee of the Affiliated Hospital of Hubei University of Chinese Medicine. Melanoma tissue samples for research were obtained after 42 of patients with melanoma provided written informed consent. All patients did not receive radiotherapy or chemotherapy. Clinical information of all recruited subjects was listed in Table 1. Melanoma specimens and adjacent normal tissues were immediately frozen in liquid nitrogen after resection and then stored at −80°C until use. Tumor tissues were confirmed as malignant melanoma by at least 2 pathologists. A375, WM35, SK-MEL-5, and SK-MEL-2 cell lines were achieved from cell bank of Wuhan University and cultured in DMEM with 10% fetal bovine serum, 100 U/mL penicillin, and 100 *μ*g/mL streptomycin (Gibco, USA) in a humidified atmosphere of 5% CO2 at 37°C. Human epidermal melanocytes (HEMa-LP) were obtained from cascade biologists, UK, and were cultured in HMGS-2 medium in the same condition as described in melanoma cell lines.

### 2.2. Plasmid Construction and Transfection

MiR-499-5p mimics, miR-499-5p inhibitors, sh-MEG3, pcDNA-MEG3, sh-CYLD, and pcDNA-CYLD were obtained from GeneCopoeia. Transfection was applied with Lipofectamine 2000 reagents (Invitrogen) on the basis of the protocol description.

### 2.3. RNA Extraction and Quantitative Real-Time PCR

TRIzol reagent was used to extract total RNA (Thermo Fisher Scientific, USA) from tissues and cells. MiR-499-5p cDNA was obtained using the TaqMan miRNA reverse transcription kit. The miR-499-5p level was quantified by qRT-PCR using TaqMan human MiRNA assay kit. The cDNA syntheses and quantitative PCR of MEG3 were applied with the SYBR PrimeScript RT-PCR kit (Takara). U6 and *β*-actin were used as the endogenous control. The 2-ΔΔCT method was utilized to measure the relative fold difference. Primers for miR-499-5p and U6 were from GeneCopoeia (HmiRQP0543, HmiRQP9001). Primers for MEG3 and *β*-actin were listed as follows: MEG3-F: 5′-CCTTCCATGCTGAGCTGCT-3′; MEG3-R: 5′-TGTTGGTGGGATCCAGGAAA-3′; *β*-actin-F: 5′-TGGACTTCGAGCAGGAAATGG-3′; *β*-actin-R: 5′-ACGTCGCACTTCATGATCGAG-3′.

### 2.4. Western Blot Assay

Tumor tissues and cells were lysed and total proteins were extracted using RIPA buffer kit according to the manufacturer's protocol. The concentration of proteins was detected with a BCA protein kit. The proteins were separated on 10% SDS-PAGE assay and moved to a PVDF membrane. The membranes were blocked with 5% milk protein in TBST and labeled with primary antibodies at 4°C overnight followed by incubation with secondary antibody for 1 h at room temperature before detection by an enhanced chemiluminescence system. The listed antibodies as primary antibodies were used to examine the corresponding protein expression: anti-CYLD (1 : 500; Abcam, UK), anti-E-cadherin (1 : 200; Abcam, UK), anti-N-cadherin (1 : 500; Abcam, UK), anti-cyclin D1 (1 : 1000; Abcam, UK), anti-*β*-actin (1 : 2000; Abcam, UK), and anti-GAPDH (1 : 1000, Santa Cruz, USA). Anti-HRP-conjugated antibody (1 : 3000; Abcam, UK) was used as the secondary antibody.

### 2.5. CCK-8 Assay

Cells proliferation was assessed by CCK-8 assay according to the manufacturer's protocol. A375 cells after transfection were incubated in 96-well plates. At 12, 24, 48, and 72 h, 90 *μ*l fresh culture media and 10 *μ*l CCK-8 solutions were added to each sample. Subsequently, transfected A375 cells were incubated at 37°C for 2 h. The optical density value was determined by a microplate reader at 450 nanometers.

### 2.6. Cell Apoptosis and Cycle Assay

The transfected cells were incubated in plates for 48 hours and stained with Annexin V-FITC and PI. Flow cytometry analysis was carried out to analyze cell apoptosis according to the manufacturer's guidelines. Propidium iodide cell cycle detection kit was conducted to analyze cell cycle.

### 2.7. EdU Assays

Melanoma cells were cultured on cover glass in 24-well plates. Cell growth was measured by an EdU incorporation assay in accordance with the product protocol. Briefly, A375 cells were cultured in DMEM with EdU labeling for 4 hours; then cells were fixed, permeated, and stained with EdU antibody. Cell nuclei were labeled by Hoechst 33342 (RiboBio, Guangzhou, China). Lastly, the treated cells were observed by laser scanning microscope.

### 2.8. Transwell Chamber Assay

The transfected A375 cells were incubated for 48 h. The cell invasion ability was assessed by Transwell assay. The upper chamber was covered with matrigel. 2 × 10^5^ cells were added to upper chamber with serum-free medium and the medium with 20% FBS was added to the lower chamber. After 48 h, cotton swab was utilized to wipe off the cells in the upper chamber. Subsequently, cells in the lower chamber were fixed and stained with 0.5% crystal violet. A microscope was applied to count the number of cells.

### 2.9. Wound-Healing Assay

Cells after transfection were cultured in appropriate condition for 48 hours. Wounds were created by a pipette tip on the cell monolayer. Cells were then washed with PBS and incubated in DMEM with 10% FBS for 48 h. Wound closure was imaged using a microscope at 0 and 48 h and measured by subtracting the final wound width from the initial wound width.

### 2.10. Luciferase Reporter Assay

The putative binding sequences of MEG3-WT, MEG3-MUT, CYLD-WT, and CYLD-MUT were, respectively, cloned into the pMIR-REPORT vectors. MiR-499-5p mimics, miR-499-5p mimics + pcDNA-control, and miR-499-5p + pcDNA-MEG3 or miR-control were transfected into A375 cells with reporter plasmids using Lipofectamine 2000 (Invitrogen). After 48 h, Dual-Luciferase Reporter Assay System (Promega) was used to detect the luciferase activity.

### 2.11. Tumor Xenograft Model

A375 cells after transfection were transplanted subcutaneously into nude mice (25–30 g, six weeks old, *n* = 6); mice were euthanized on 5 d, 10 d, 15 d, 20 d, and 25 d, and the weight and volume of tumor tissues were assessed and recorded.

### 2.12. Chemosensitivity Assay

A375 cells after transfection were cultured in fresh medium and then seeded into 96-well plates. Various doses of cisplatin or 5-FU were added to the cell culture for 72 h. Next, the proliferation of cell was evaluated using CCK-8 assay.

### 2.13. Statistical Analysis

SPSS 16.0 software was applied for statistical analysis including *t*-test, one-way analysis of variance with post hoc contrasts by Student-Newman-Keuls test, Chi-squared test, Pearson correlation analysis, and Kaplan-Meier plot. Data were showed as the mean ± standard deviation. *p* < 0.05 was regarded as statistically significant difference.

## 3. Results

### 3.1. The Expression of MEG3 and CYLD Was Decreased, but the Expression of miR-499-5p Was Increased in Melanoma Specimens and Cell Lines

**Figure 1:**
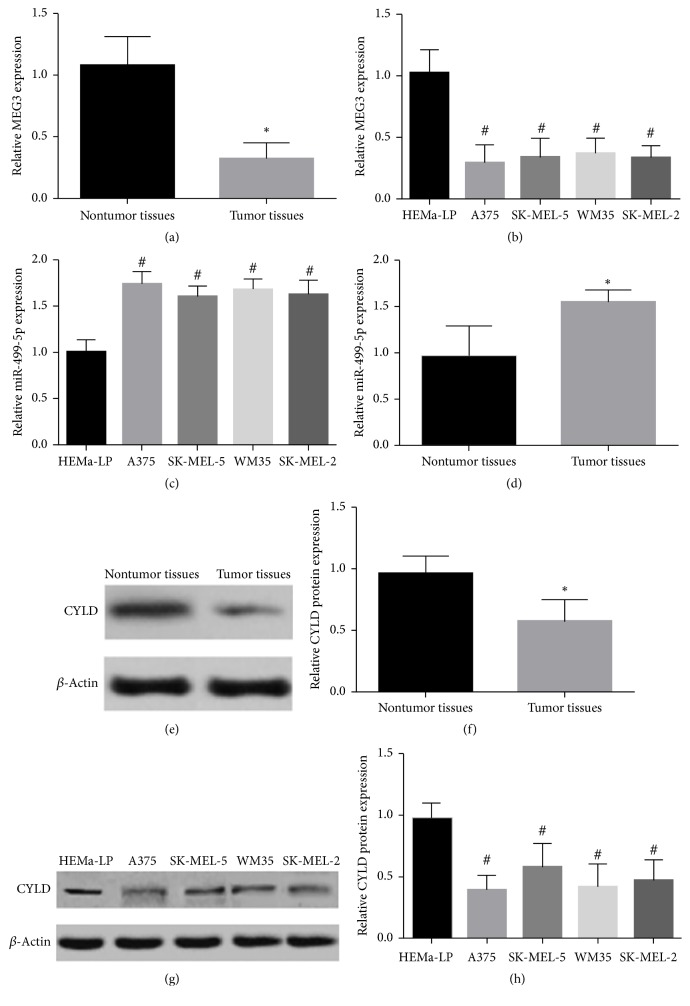
The levels of MEG3, miR-499-5p, and CYLD in melanoma samples and cell lines. (a) The MEG3 expression was reduced markedly in 42 melanoma samples in comparison with paired normal samples. (b) Reduced expression of MEG3 was found in melanoma cell lines in comparison with normal skin melanocytes (HEMa-LP). (c) The expression of miR-499-5p in melanoma cell lines was distinctly higher, respectively, than in normal human melanocytes (HEMa-LP). (d) The level of miR-499-5p was greatly overexpressed in tumor tissue samples in comparison with nontumor samples. (e and f) The CYLD level was decreased markedly in tumor samples in comparison with nontumor samples. (g) The CYLD expression in melanoma cell lines was considerably lower than in normal human melanocytes (HEMa-LP). (^*∗*^*p* < 0.05 compared with nontumor tissues group. ^#^*p* < 0.05 compared with HEMa-LP group.)

**Figure 2:**
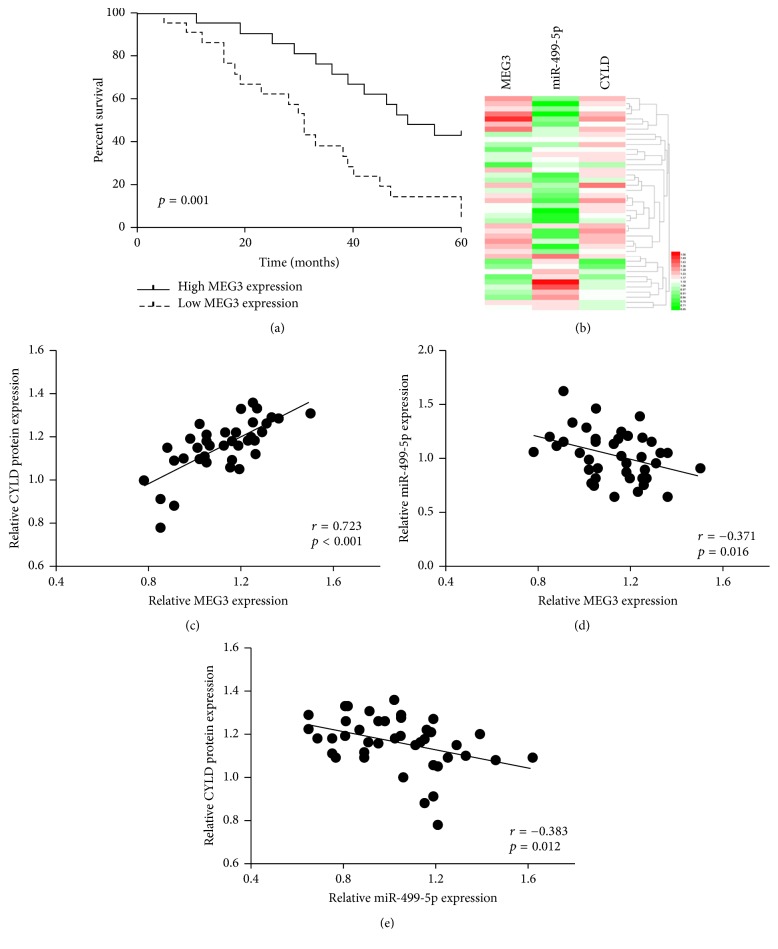
The relationship between the MEG3 level and prognosis of individuals with melanoma and the correlations among MEG3, miR-499-5p, and CYLD in melanoma tissues.(a) Low MEG3 expression was related to decreased overall survival. (b) The heatmap showed that the MEG3 and CYLD levels were negatively related to the level of miR-499-5p. (c–e) Pearson correlation analysis was used to assess the correlations among MEG3, miR-499-5p, and CYLD in melanoma tissues.

qRT-PCR and western blot assay were applied for detecting the expression of MEG3, miR-499-5p, and CYLD. It was identified that the level of MEG3 and CYLD was significantly decreased, but the expression of miR-499-5p was dramatically increased in melanoma tissues and cell lines compared with paired nontumor tissues or HEMa-LP cells (Figure 1). The participants were split into high MEG3 level group (*n* = 21) and low MEG3 level group (*n* = 21) in accordance with the median value of MEG3. No distinct association was found among the MEG3 level, age, and sex in individuals with melanoma, but the downregulation of MEG3 was markedly associated with tumor thickness, TNM stage, lymph node involvement, and distant metastasis of malignant melanoma as showed in Table 1. In addition, reduced expression of MEG3 was positively associated with decreased overall survival (Figure 2(a)). The correlations were confirmed by Pearson correlation analysis among MEG3, miR-499-5p, and CYLD (Figures 2(b)–2(e)).

### 3.2. Upregulation of MEG3 Inhibited A375 Cells Proliferation, Induced Cell Apoptosis, and Retarded the Metastasis of A375 Cells

**Figure 3:**
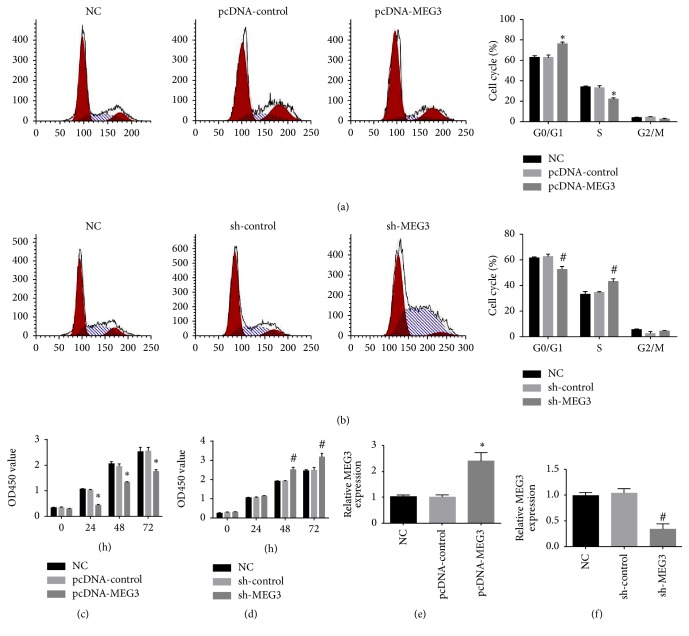
The effect of MEG3 on A375 cell proliferation and cell cycle. (a) Upregulation of MEG3 effectively blocked cell cycle at the G0/G1 stage. (b) Downregulation of MEG3 heavily promoted cell cycle. (c) The transfection of pcDNA-meg3 attenuated the proliferation of A375 cells. (d) The growth of A375 cells was enhanced significantly in sh-MEG3 group. (e) The MEG3 level was increased in pcDNA-MEG3 group. (f) The expression of MEG3 was decreased in sh-MEG3 group. (^*∗*^*p* < 0.05 compared with NC group and pcDNA-control group. ^#^*p* < 0.05 compared with NC group and sh-control group.)

**Figure 4:**
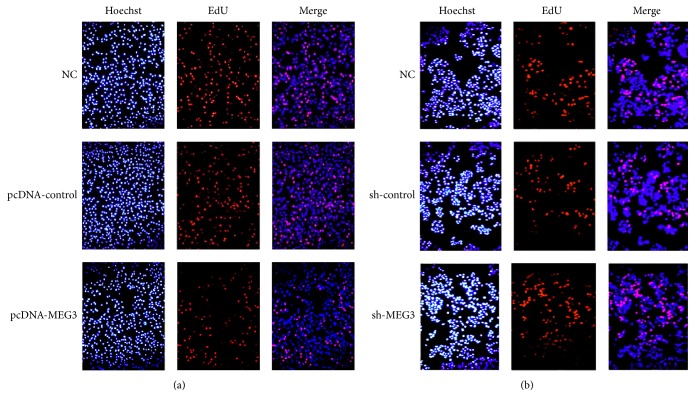
The effect of MEG3 on A375 cell cycle detected by immunofluorescence analysis. (a) Overexpression of MEG3 decreased markedly the percentage of cells at S phase. (b) Reduced expression of MEG3 increased significantly the percentage of cells at S stage.

**Figure 5:**
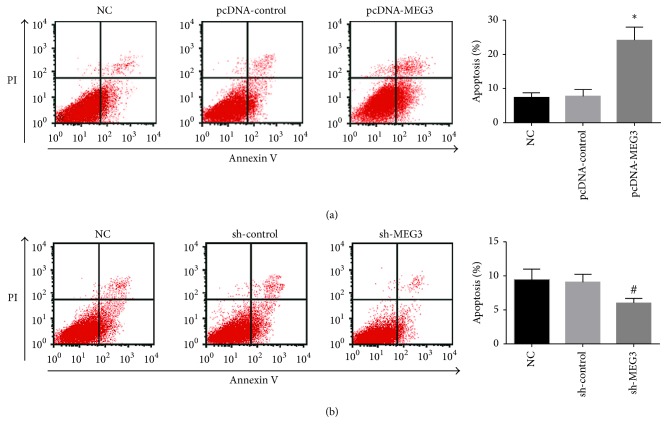
The effect of MEG3 on A375 cells apoptosis. (a) Upregulation of MEG3 enhanced significantly the apoptosis of A375 cells. (b) Downregulation of MEG3 inhibited distinctly A375 cells apoptosis (^*∗*^*p* < 0.05 compared with NC group and pcDNA-control group; ^#^*p* < 0.05 compared with NC group and sh-control group).

**Figure 6:**
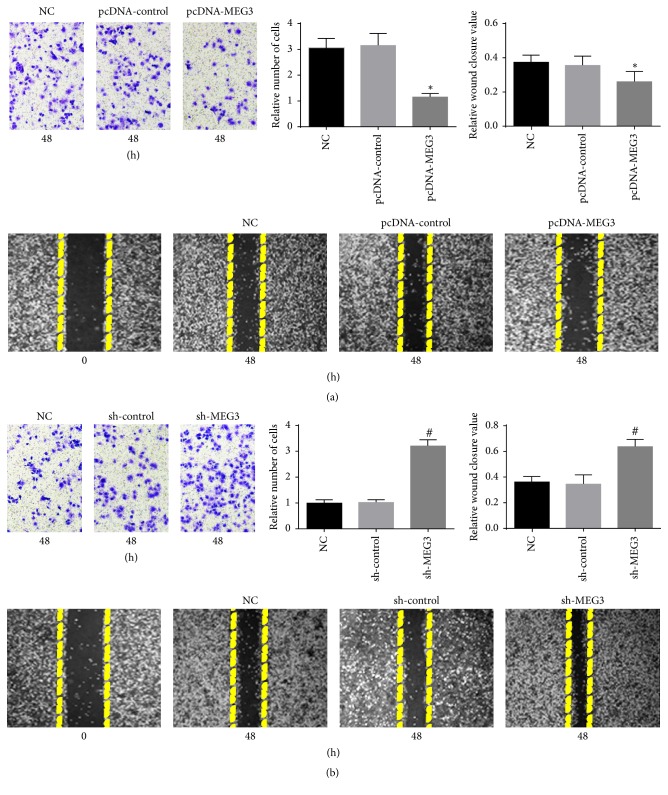
MEG3 affected A375 cells migration and invasion in vitro. (a) Transwell assay and wound-healing assay were used to assess the influence of pcDNA-MEG3 on migration and invasion of A375 cells. Upregulation of MEG3 markedly retarded the metastasis of A375 cells in pcDNA-MEG3 group compared with normal control group and pcDNA-control group. (b) sh-MEG3 enhanced distinctly the invasion and migration of A375 cells in sh-MEG3 group in comparison with normal control group and sh-MEG3 group. (^*∗*^*p* < 0.05 compared with NC group and pcDNA-control group. ^#^*p* < 0.05 compared with NC group and sh-control group.)

To explore the impacts of MEG3 on the growth and metastasis of melanoma, A375 cells were transfected with pcDNA-MEG3. qRT-PCR assay revealed that the level of MEG3 in A375 cells was distinctly increased in pcDNA-MEG3 group compared with normal control group and pcDNA-control group (Figure 3(e)). Our results showed that A375 cell growth was inhibited in pcDNA-MEG3 group in comparison with normal control group and pcDNA-control group (Figure 3(c)). Cell cycle assay showed that a larger percentage of A375 cells in pcDNA-MEG3 group were retarded at the G0/G1 stage compared with normal control group and pcDNA-control group (Figures 3(a) and 4(a)). Furthermore, A375 cells apoptosis was significantly enhanced, and the invasion and migration of A375 cells were distinctly inhibited in pcDNA-MEG3 group compared with normal control group and pcDNA-control group (Figures 5(a) and 6(a)). On the contrary, the level of MEG3 was significantly reduced in A375 cells after transfection with sh-MEG3 (Figure 3(f)). Reduced expression of MEG3 inhibited A375 cells apoptosis and enhanced A375 cells cycle, proliferation, and invasion (Figures 3(b), 3(d), 4(b), 5(b), and 6(b)). Thus, we concluded that overexpression of MEG3 could inhibit melanoma cells growth and reduce cell invasion.

### 3.3. MEG3 Sponged Directly miR-499-5p in A375 Cells

**Figure 7:**
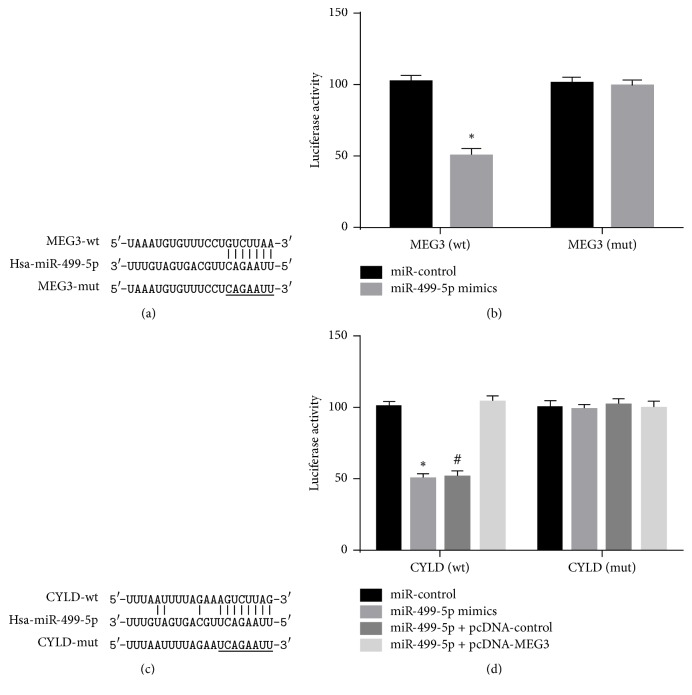
Bioinformatics analysis demonstrated the putative binding region of MEG3, miR-499-5p, and CYLD mRNA. (a) MIR-499-5p and its putative binding sequence for wild type MEG3. (b) miR-499-5p mimics inhibited luciferase reporter activities of MEG3 in wild type but did not affect that of MEG3 in mutated type. (c) MIR-499-5p and its predicted binding sites for WT in CYLD. (d) Luciferase reporter gene assay results. (^*∗*^*p* < 0.05 compared with miR-control group. ^#^Compared with miR-499-5p + pcDNA-MEG3 group.)

Bioinformatics analysis showed miR-499-5p may be a target of MEG3. Luciferase assay was performed to ascertain whether MEG3 could regulate miR-499-5p expression by acting as molecular sponge. The result manifested that miR-499-5p mimics dramatically alleviated the luciferase activity of MEG3-wt but had no significant effect on that of MEG3-mut (Figures 7(a) and 7(b)).

### 3.4. MiR-499-5p Directly Targeted CYLD in A375 Cells

Bioinformatics analysis manifested that miR-499-5p could bind directly to 3′-UTR-CYLD. Luciferase assay manifested that miR-499-5p mimics suppressed obviously the luciferase activity of CYLD-wt, whereas they did not significantly affect the luciferase expression of CYLD-mut. Furthermore, the cotransfection of miR-499-5p mimics with pcDNA-MEG3 did not have significant influence on the luciferase activity in CYLD-wt and CYLD-mut. Though the cotransfection of miR-499-5p mimics with pcDNA-control reduced luciferase expression of CYLD-wt, it did not make much difference on the luciferase activity in CYLD-mut (Figures 7(c) and 7(d)).

### 3.5. MEG3 Reinforced the Expression of CYLD by Sponging miR-499-5p

**Figure 8:**
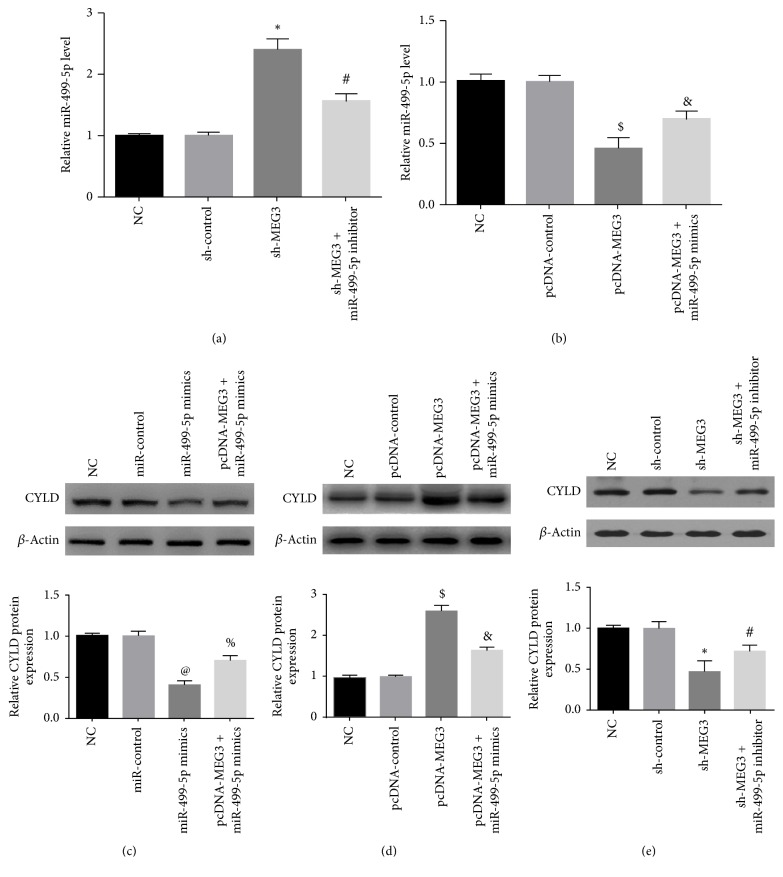
MEG3 modulated the expression of CYLD by sponging miR-499-5p in A375 cells. (a, b) Reduced expression of MEG3 increased the miR-499-5p level; oppositely, overexpression of MEG3 decreased the miR-499-5p level. (c) Upregulation of miR-499-5p suppressed the expression of CYLD in A375 cells, whereas the cotransfection of miR-499-5p mimics with pcDNA-MEG3 attenuated the inhibitory function of miR-499-5p on the expression of CYLD in A375 cells. (d) Upregulation of MEG3 promoted the expression of CYLD, but the enhancing function of pcDNA-MEG3 was limited by the cotransfection of miR-499-5p mimics with pcDNA-MEG3 in A375 cells. (e) Downregulation of MEG3 markedly suppressed the expression of CYLD, but miR-499-5p inhibitors alleviated the impeding function of sh-MEG3 on the expression of CYLD in A375 cells. (^*∗*^*p* < 0.05 compared with NC group and sh-control group. ^#^*p* < 0.05 compared with sh-MEG3 group. ^$^*p* < 0.05 compared with NC group and pcDNA-control group. ^&^*p* < 0.05 compared with pcDNA-MEG3 group. ^@^*p* < 0.05 compared with NC group and miR-control group. ^%^*p* < 0.05 compared with miR-499-5p mimics group.)

The transfection of sh-MEG3 or pcDNA-MEG3 regulated the expression of miR-499-5p. Overexpression of MEG3 dramatically decreased the level of miR-499-5p and promoted CYLD protein expression; however, downregulation of MEG3 distinctly increased the miR-499-5p expression and inhibited CYLD protein expression. Meanwhile, the cotransfection of miR-499-5p mimics with pcDNA-MEG3 abrogated remarkably the promotion effect of pcDNA-MEG3 on CYLD protein expression. The cotransfection of miR-499-5p inhibitors with sh-MEG3 alleviated greatly the inhibitory function of sh-MEG3 on the expression of CYLD (Figure 8).

### 3.6. MEG3 Acted as an Antitumor Factor via Regulating the Expression of CYLD in Melanoma

**Figure 9:**
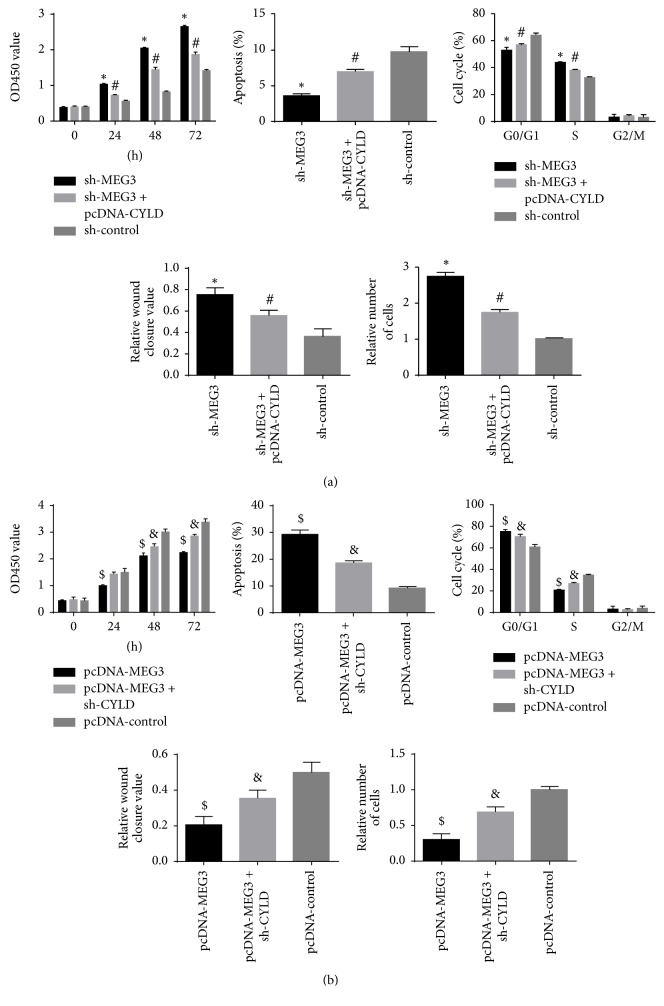
MEG3 inhibited the growth and invasion of A375 cells by regulating the expression of CYLD. (a) Downregulation of MEG3 enhanced the growth, cell cycle, and invasion of A375 cells and suppressed the apoptosis of A375 cells compared with sh-control group. Besides, forced expression of CYLD attenuated the promoting function of sh-MEG3 in the growth, cell cycle, and invasion of A375 cells and the suppressing function of sh-MEG3 in the apoptosis of A375 cells. (b) Upregulation of MEG3 attenuated the proliferation, cell cycle, and invasion of A375 cells and promoted A375 cells apoptosis compared with pcDNA-control group. In addition, reduced expression of CYLD enhanced the proliferation, cell cycle, and invasion of A375 cells transfected with pcDNA-MEG3 and reduced the apoptosis of A375 cells transfected with pcDNA-MEG3. (^*∗*^*p* < 0.05 compared with sh-control group. ^#^*p* < 0.05 compared with sh-MEG3 group. ^$^*p* < 0.05 compared with pcDNA-control group. ^&^*p* < 0.05 compared with pcDNA-MEG3 group.)

To further investigate whether CYLD mediated the function of MEG3 in the progression of melanoma, sh-control, sh-MEG3, and sh-MEG3 + pcDNA-CYLD were transfected into A375 cells, respectively. As a consequence, sh-MEG3 promoted the growth and metastasis of A375 cells and inhibited cell apoptosis, whereas the cotransfection of pcDNA-CYLD abrogated the promoting function of sh-MEG3 on the proliferation and metastasis ability of A375 cells (Figure 9(a)). Conversely, after A375 cells were transfected with pcDNA-control, pcDNA-MEG3, or pcDNA-MEG3+sh-CYLD separately, we identified that the transfection of pcDNA-MEG3 suppressed the growth, invasion, and migration of A375 cells, but sh-CYLD alleviated the inhibiting function of pcDNA-MEG3 in A375 cells (Figure 9(b)). Therefore, the function of MEG3 in growth and metastasis of A375 cells was mediated, at least in part, by CYLD.

### 3.7. MEG3 Enhanced E-Cadherin Expression and Suppressed N-Cadherin and Cyclin D1 Expression via Regulating the Expression of CYLD in A375 Cells

**Figure 10:**
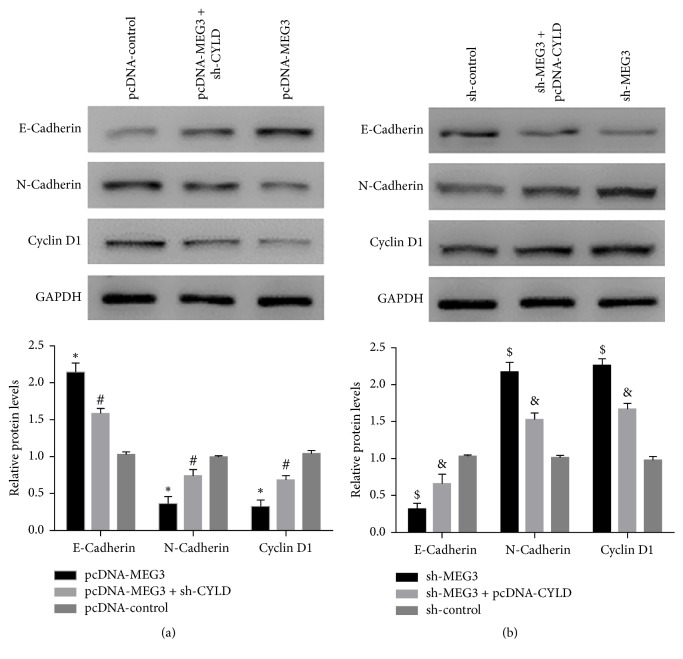
MEG3 modulated the expression of E-cadherin, N-cadherin, and cyclin D1 by regulating the expression of CYLD in A375 cells. (a) Forced expression of MEG3 enhanced the E-cadherin level and impeded the expression of N-cadherin and cyclin D1 in comparison with pcDNA-control group, but the cotransfection of sh-CYLD limited the function of pcDNA-MEG3 in these proteins in A375 cells. (b) Downregulation of MEG3 reduced the E-cadherin level and elevated the level of N-cadherin and cyclin D1 in comparison with sh-control group, whereas forced expression of CYLD reversed the effect of sh-MEG3 on these proteins expression in A375 cells. (^*∗*^*p* < 0.05 compared with pcDNA-control group. ^#^*p* < 0.05 compared with pcDNA-MEG3 group. ^$^*p* < 0.05 compared with sh-control group. ^&^*p* < 0.05 compared with sh-MEG3 group.)

To further explore the underlying mechanism of MEG3 on inhibiting the growth and invasion of A375 cells, molecules related to EMT processing and cell cycle were evaluated by western blot assay. Overexpression of MEG3 remarkably strengthened the level of E-cadherin and alleviated the level of N-cadherin and cyclin D1, while cotransfection of pcDNA-MEG3 with sh-CYLD abrogated the function of MEG3 in the listed proteins expression. Conversely, reduced expression of MEG3 suppressed E-cadherin expression and promoted the expression of N-cadherin and cyclin D1, but pcDNA-CYLD attenuated the effect of sh-MEG3 on the expression of the above proteins in A375 cells (Figure 10).

### 3.8. Upregulation of MEG3 Suppressed Melanoma Growth in an Animal Experiment and Improved Chemosensitivity in A375 Cells

**Figure 11:**
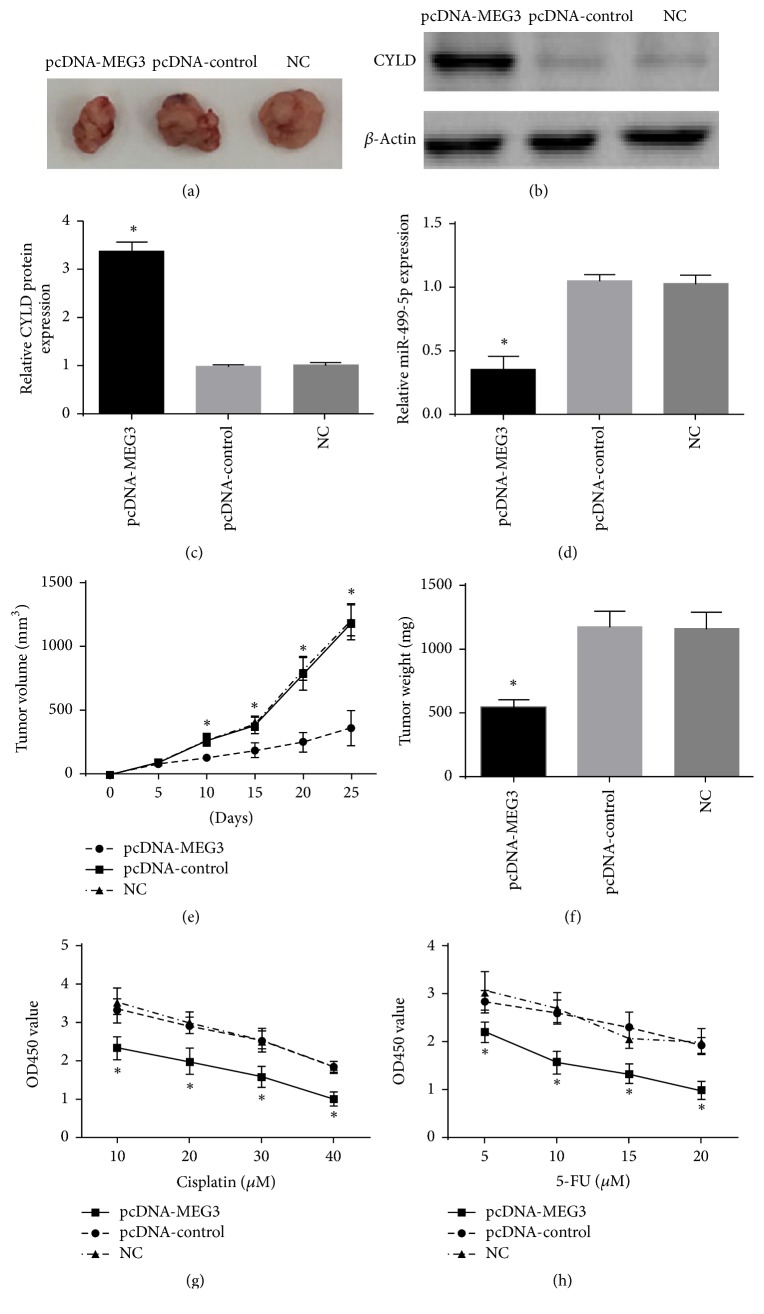
Forced expression of MEG3 impeded melanoma growth and improved chemosensitivity of A375 cells to cisplatin or 5-FU. (a) Xenograft tumor tissues in pcDNA-MEG3 group were significantly smaller than in pcDNA-control group and NC group. (b-c) The CYLD level in xenograft tumor was increased distinctly in pcDNA-MEG3 group. (d) The miR-499-5p level in xenograft tumor was dramatically reduced in pcDNA-MEG3 group. (e-f) Upregulation of MEG3 decreased the tumor volume and weight. (g-h) Forced expression of MEG3 enhanced chemosensitivity of A375 cells to cisplatin or 5-FU treatment. (^*∗*^*p* < 0.05 compared with pcDNA-control group and NC group.)

To identify that in vivo MEG3 could regulate melanoma growth, A375 cells transfected with pcDNA-MEG3 were implanted subcutaneously into nude mouse. The volume of xenograft tumor was significantly smaller in pcDNA-MEG3 group than in normal control group and pcDNA-control group at 10 d, 15 d, 20 d, and 25 d after transplantation. The final weight of xenograft tumor was dramatically reduced in pcDNA-MEG3 group compared with normal control group and pcDNA-control group. In addition, the level of miR-499-5p in xenograft tumor was decreased markedly and the CYLD level in xenograft tumor was dramatically elevated in pcDNA-MEG3 group compared with normal control group and pcDNA-control group. Finally, A375 cells were treated with 5-FU or cisplatin, respectively, in pcDNA-MEG3 group, pcDNA-control group, and normal control group. Subsequently, CCK-8 assay showed that the growth of A375 cells was more suppressed in pcDNA-MEG3 group in comparison with normal control group and pcDNA-control group (Figure 11).

## 4. Discussion

lncRNA represents a type of transcript that is more than 200 nucleotides in length and has little or no protein coding capacity [20]. Numerous lncRNAs were found to function as antitumor or protumor factors in diverse cancers [21, 22]. In this research, we confirmed that MEG3 played antitumor role in malignant melanoma development through miR-499-5p/CYLD axis.

In the report, we confirmed that MEG3 expression was distinctly decreased in 42 of melanoma samples and in melanoma cell lines. Meanwhile, low expression of MEG3 was positively correlated with poor prognosis in patients with melanoma. In in vitro and in vivo assays, upregulation of MEG3 inhibited distinctly the growth and metastasis of A375 cells, enhanced A375 cell apoptosis, and retarded cell cycle at G0/G1 stage. In addition, overexpression of MEG3 lessened significantly xenograft tumor volume and weight. Collectively, these results concluded that MEG3 played a tumor suppressor role in the tumorigenesis of melanoma.

Current researches have identified that lncRNAs can act as a sponge to competitively target miRNA and modulate the function of the targeting miRNA [23]. With the aid of bioinformatics online tools and luciferase reporter assay, miR-499-5p was found as the downstream target of MEG3. In addition, we forecasted and confirmed that CYLD acted as the binding mRNA of miR-499-5p. Further analysis validated that the level of miR-499-5p was inversely associated with CYLD level and MEG3 expression in melanoma tissues and cell lines. Finally, the rescue experiment strongly identified that MEG3 controlled the level of CYLD by targeting miR-499-5p.

**Figure 12:**
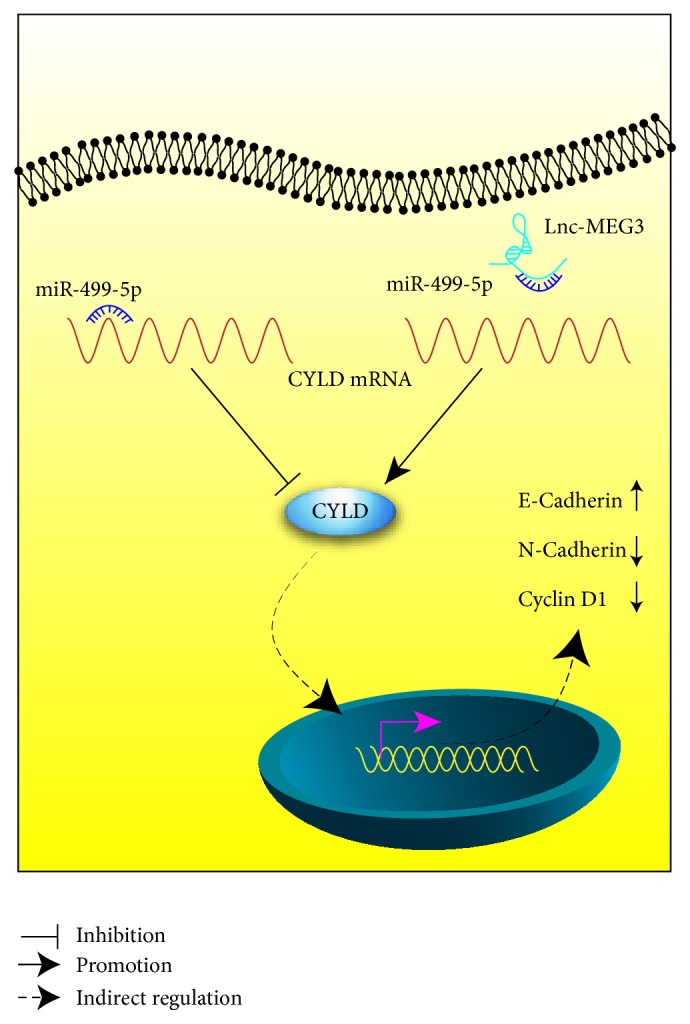
The diagram illustrated the molecular regulatory mechanism of MEG3 in the tumorigenesis of melanoma.

CYLD has been reported as an important antitumor gene in numerous malignant tumors. For example, inhibition of CYLD expression enhanced human malignant melanoma growth and invasion [24]. Increased CYLD suppressed the development of hepatocellular carcinoma [25] and gastric cancer [26]. Our results showed that upregulation of CYLD alleviated the effect of sh-MEG3 on the growth, apoptosis, and migration of A375 cells, and downregulation of CYLD attenuated the function of pcDNA-MEG3 in cell proliferation, cell apoptosis, and metastasis of A375 cells. Furthermore, western blot assay confirmed that MEG3 modulated the EMT markers and cyclin D1 expression by regulating the expression of CYLD in A375 cells. The result was consistent with the previous reports [15, 27]. Taken together, CYLD mediated, at least in part, the effect of MEG3 on tumorigenesis of melanoma (Figure 12).

A xenograft model was used to further investigate the effect of MEG3 on melanoma in vivo. We found that pcDNA-MEG3 distinctly decreased the weight and volume of tumors and improved chemosensitivity of A375 cells to cisplatin or 5-FU treatment.

Together, this report emphasizes that MEG3 acts as an antitumor lncRNA for malignant melanoma by regulating miR-499-5p/CYLD axis; MEG3 may be a novel and crucial biomarker in patients with melanoma and can be used as a promising molecular target in the treatment of malignant melanoma. Nevertheless, it should be pointed out that the effects of MEG3 need be further investigated on more melanoma cell lines other than A375 cells and the underlying mechanism of MEG3 demands intensive elucidation on promoting A375 cell apoptosis.

## Figures and Tables

**Table 1:** The association between clinical features and the MEG3 level in individuals with malignant melanoma.

Clinicopathological finding	Patients	MEG3		*p* value
	*N* = 42	High	Low	
Age (years)				
≦60	25	13	12	0.753
>60	17	8	9	
Sex				
Male	21	12	9	0.355
Female	21	9	12	
Tumor thickness (mm)				
≤1	16	12	4	0.011
>1	26	9	17	
TNM classification				
I + II	17	13	4	0.005
III + IV	25	8	17	
Lymph node involvement				
No	19	16	3	0.000
Yes	23	5	18	
Distant metastasis				
No	27	19	8	0.000
Yes	15	2	13	

## References

[1] Trotter S. C., Sroa N., Winkelmann R. R., Olencki T., Bechtel M. (2013). A global review of melanoma follow-up guidelines. *Journal of Clinical and Aesthetic Dermatology*.

[2] Clarke C. A., McKinley M., Hurley S. (2017). Continued Increase in Melanoma Incidence across all Socioeconomic Status Groups in California, 1998–2012. *Journal of Investigative Dermatology*.

[3] Luo C., Shen J. (2017). Research progress in advanced melanoma. *Cancer Letters*.

[4] Mehra M., Chauhan R. (2017). Long Noncoding RNAs as a Key Player in Hepatocellular Carcinoma. *Biomarkers in Cancer*.

[5] Sun T. (2018). Long noncoding RNAs act as regulators of autophagy in cancer. *Pharmacological Research*.

[6] Chi H.-C., Tsai C.-Y., Tsai M.-M., Yeh C.-T., Lin K.-H. (2017). Roles of long noncoding rnas in recurrence and metastasis of radiotherapy-resistant cancer stem cells. *International Journal of Molecular Sciences*.

[7] Bhan A., Soleimani M., Mandal S. S. (2017). Long noncoding RNA and cancer: A new paradigm. *Cancer Research*.

[8] He Y., Luo Y., Liang B., Ye L., Lu G., He W. (2017). Potential applications of MEG3 in cancer diagnosis and prognosis. *Oncotarget *.

[9] Li J., Zi Y., Wang W. (2017). LncRNA MEG3 inhibits cell proliferation and metastasis in chronic myeloid leukemia via targeting MiR-184. *Oncology Research*.

[10] Zhang C.-Y., Yu M.-S., Li X., Zhang Z., Han C.-R., Yan B. (2017). Overexpression of long non-coding RNA MEG3 suppresses breast cancer cell proliferation, invasion, and angiogenesis through AKT pathway. *Tumor Biology*.

[11] Zhang W., Shi S., Jiang J., Li X., Lu H., Ren F. (2017). LncRNA MEG3 inhibits cell epithelial-mesenchymal transition by sponging miR-421 targeting E-cadherin in breast cancer. *Biomedicine & Pharmacotherapy*.

[12] Tauriello D. V. F., Haegebarth A., Kuper I. (2010). Loss of the Tumor Suppressor CYLD Enhances Wnt/*β*-Catenin Signaling through K63-Linked Ubiquitination of Dvl. *Molecular Cell*.

[13] Urbanik T., Koehler B. C., Wolpert L. (2014). CYLD deletion triggers nuclear factor-KB-signaling and increases cell death resistance in murine hepatocytes. *World Journal of Gastroenterology*.

[14] Lim J. H., Jono H., Komatsu K. (2012). CYLD negatively regulates transforming growth factor-*β*-signalling via deubiquitinating Akt. *Nature Communications*.

[15] Ke H., Augustine C. K., Gandham V. D. (2013). CYLD inhibits melanoma growth and progression through suppression of the jnk/ap-1 and *β*1-integrin signaling pathways. *Journal of Investigative Dermatology*.

[16] Krutzfeldt J. (2016). Strategies to use microRNAs as therapeutic targets. *Best Practice & Research Clinical Endocrinology & Metabolism*.

[17] Ross C. L., Kaushik S., Valdes-Rodriguez R., Anvekar R. MicroRNAs in cutaneous melanoma: Role as diagnostic and prognostic biomarkers. *Journal of Cellular Physiology*.

[18] Li M., Zhang S., Wu N., Wu L., Wang C., Lin Y. (2016). Overexpression of miR-499-5p inhibits non-small cell lung cancer proliferation and metastasis by targeting VAV3. *Scientific Reports*.

[19] Liu X., Zhang Z., Sun L. (2011). MicroRNA-499-5p promotes cellular invasion and tumor metastasis in colorectal cancer by targeting FOXO4 and PDCD4. *Carcinogenesis*.

[20] Ling H., Fabbri M., Calin G. A. (2013). MicroRNAs and other non-coding RNAs as targets for anticancer drug development. *Nature Reviews Drug Discovery*.

[21] Wu X., Zhang P., Zhu H., Li S., Chen X., Shi L. (2017). Long noncoding RNA FEZF1-AS1 indicates a poor prognosis of gastric cancer and promotes tumorigenesis via activation of Wnt signaling pathway. *Biomedicine & Pharmacotherapy*.

[22] Hu J., Song C., Duan B. (2017). LncRNA-SVUGP2 suppresses progression of hepatocellular carcinoma. *Oncotarget *.

[23] Liang R., Han B., Li Q., Yuan Y., Li J., Sun D. (2017). Using RNA sequencing to identify putative competing endogenous RNAs (ceRNAs) potentially regulating fat metabolism in bovine liver. *Scientific Reports*.

[24] Zhang K., Guo L. (2018). MiR-767 promoted cell proliferation in human melanoma by suppressing CYLD expression. *Gene*.

[25] Ni F., Zhao H., Cui H. (2015). MicroRNA-362-5p promotes tumor growth and metastasis by targeting CYLD in hepatocellular carcinoma. *Cancer Letters*.

[26] Zhu M., Zhou X., Du Y. (2016). MiR-20a induces cisplatin resistance of a human gastric cancer cell line via targeting CYLD. *Molecular Medicine Reports*.

[27] Qiu H., Yuan S., Lu X. (2016). miR-186 suppressed CYLD expression and promoted cell proliferation in human melanoma. *Oncology Letters*.

